# Artificial Intelligence–Based Co-Facilitator (AICF) for Detecting and Monitoring Group Cohesion Outcomes in Web-Based Cancer Support Groups: Single-Arm Trial Study

**DOI:** 10.2196/43070

**Published:** 2024-07-22

**Authors:** Yvonne W Leung, Elise Wouterloot, Achini Adikari, Jinny Hong, Veenaajaa Asokan, Lauren Duan, Claire Lam, Carlina Kim, Kai P Chan, Daswin De Silva, Lianne Trachtenberg, Heather Rennie, Jiahui Wong, Mary Jane Esplen

**Affiliations:** 1 de Souza Institute University Health Network Toronto, ON Canada; 2 Department of Psychiatry Temerty Faculty of Medicine University of Toronto Toronto, ON Canada; 3 College of Professional Studies Northeastern University Toronto, ON Canada; 4 Centre for Data Analytics and Cognition La Trobe University Melbourne Australia; 5 Centre for Psychology and Emotional Health Toronto, ON Canada; 6 BC Cancer Agency Vancouver, BC Canada

**Keywords:** group cohesion, LIWC, online support group, natural language processing, NLP, emotion analysis, machine learning, sentiment analysis, emotion detection, integrating human knowledge, emotion lining, cancer, oncology, support group, artificial intelligence, AI, therapy, online therapist, emotion, affect, speech tagging, speech tag, topic modeling, named entity recognition, spoken language processing, focus group, corpus, language, linguistic

## Abstract

**Background:**

Commonly offered as supportive care, therapist-led online support groups (OSGs) are a cost-effective way to provide support to individuals affected by cancer. One important indicator of a successful OSG session is group cohesion; however, monitoring group cohesion can be challenging due to the lack of nonverbal cues and in-person interactions in text-based OSGs. The Artificial Intelligence–based Co-Facilitator (AICF) was designed to contextually identify therapeutic outcomes from conversations and produce real-time analytics.

**Objective:**

The aim of this study was to develop a method to train and evaluate AICF’s capacity to monitor group cohesion.

**Methods:**

AICF used a text classification approach to extract the mentions of group cohesion within conversations. A sample of data was annotated by human scorers, which was used as the training data to build the classification model. The annotations were further supported by finding contextually similar group cohesion expressions using word embedding models as well. AICF performance was also compared against the natural language processing software Linguistic Inquiry Word Count (LIWC).

**Results:**

AICF was trained on 80,000 messages obtained from Cancer Chat Canada. We tested AICF on 34,048 messages. Human experts scored 6797 (20%) of the messages to evaluate the ability of AICF to classify group cohesion. Results showed that machine learning algorithms combined with human input could detect group cohesion, a clinically meaningful indicator of effective OSGs. After retraining with human input, AICF reached an *F*_1_-score of 0.82. AICF performed slightly better at identifying group cohesion compared to LIWC.

**Conclusions:**

AICF has the potential to assist therapists by detecting discord in the group amenable to real-time intervention. Overall, AICF presents a unique opportunity to strengthen patient-centered care in web-based settings by attending to individual needs.

**International Registered Report Identifier (IRRID):**

RR2-10.2196/21453

## Introduction

### Overview

Web-based care has become increasingly important in health care delivery as a means to accessibly reduce emotional distress. Online support groups (OSG) offer a convenient solution to those who cannot attend in-person support groups [1-3]. Professionally led OSGs occur in real time with participants engaging with a therapist and other participants in the group. Therapists facilitate the sharing of personal experiences to foster a mutually supportive environment. OSG participants report an increased sense of empowerment and control, as well as improved knowledge about their conditions [4].

Cancer Chat Canada (CCC) offers web-based professionally led, synchronous, text-based support groups to patients with cancer or caregivers across 6 Canadian provinces with a text-based nature allowing for anonymity while reaching people in rural areas. All groups provided via CCC are manual-based, consisting of 8-12 sessions. Each session focuses on a specific theme, homework readings, and web-based activities. Participants exchanged their experiences and ideas through a chatbox on the CCC platform. During sessions, therapists facilitate discussions based on the weekly readings, address issues or concerns, attend to emergent emotional needs of the members, and employ therapeutic techniques that promote a continuous sense of mutual support among the 6-10 members [5].

For group interventions to be effective, therapists encourage authentic emotional expression while effectively monitoring and addressing signals of distress [6]. However, the absence of visual cues, along with the simultaneous entries by multiple participants, can impose challenges for therapists to attend to all participants’ needs during the session [4]. Therapists’ failure to recognize and respond to participants’ expressions of distress can reduce the participants’ perceived level of support, safety, and trust in the group, leading to disengagement and attrition [7].

One way to reduce attrition and improve OSG services is through tracking and monitoring group cohesion [8,9]. A cohesive group experiences a sense of warmth, comfort, acceptance, affiliation, and support from other members they value [5]. Group cohesion is associated with positive participant outcomes, including reductions in distress and improvements in interpersonal functioning [5].

Traditionally, group cohesion is measured by participant self-report instruments, such as the Harvard Community Health Plan Group Cohesiveness Scale[10] and the Group Cohesion Scale Revised [10]. Alternatively, it can be measured by content analysis, where analysts assign ratings to the participants’ statements [11]. While useful, these approaches have limitations of participant recall bias, measurement fatigue in self-reports, and time and cost of labor in post hoc qualitative analyses.

Previous studies demonstrate that a higher frequency of first-person singular pronouns use (ie, I, my), also referred to as “iTalk” or self-referential language, is a linguistic marker of general distress and is associated with negative psychological outcomes such as depression and suicidal behaviors [12-14]. In contrast, collective identity language use (ie, our group, us) was instrumental to group attachment [15]; with greater uses of references to the group as a whole and to other members predicting reduced symptoms of grief [11]. Aside from content analysis, such as Psychodynamic Work, Object Rating System [16], many studies adopted computerized textual analysis systems such as dtSearch [17], Linguistic Inquiry, and Word Count (LIWC) to track levels of cohesion through text [18-21]. In particular, Lieberman et al [20] detected group cohesion by combining LIWC to count the proportion of group referential language use and dtSearch to count words indicative of positive connotations (ie, hope, altruistic, accept, affection) within 10 words of such group referential language in an OSG for patients with Parkinson. However, Alpers et al [19] questioned the software’s ability to process complex communications, suggesting that future studies should develop systems that analyze the context of discourse for real-time analysis.

Given the evidence, group cohesion can be systematically measured by a well-designed computer analytical system. We designed the Artificial Intelligence–based Co-Facilitator (AICF) to contextually identify therapeutic outcomes, including group cohesion from conversations, and produce real-time analytics [22-25]. AICF can track basic emotions, including joy, sadness, anger, trust, fear, anticipation, disgust, surprise, and psychological outcomes such as distress, group cohesion, and hopelessness for each participant in the OSGs [22,23]. AICF extracted emotions from the text by parsing through over 120,000 lines of chat messages from a training data set to multiple levels of granularity: word, phrase, sentence, post, and posts by each user [26]. AICF employed several natural language processing (NLP) techniques, such as Word2Vec [27] and text classification models. Classification models were trained to classify posts containing group cohesion mentions to determine the level of group cohesion in this web-based conversation setting. Each level of extraction served as an input for calibration for the subsequent extraction to increase accuracy [26,28,29]. AICF could, therefore, track and inform facilitators of each participant’s level of cohesive statement use in their posts.

We hypothesized that AICF could detect first-person plural pronoun use (eg, we, our) in OSGs and group-references language use (“we-talk”) as group cohesion, machine learning–based NLP could also identify a broader definition of group cohesion, including expressing gratitude, mutual support, and sense of belonging.

### Objective

This study is focused on the development of a method to train and evaluate AICF’s ability to detect group cohesion among cancer OSG members.

## Methods

### AICF Training and Development

The steps involved in the training and development of AICF’s cohesion detection is outlined below.

#### Collecting Design Specifications

Experienced CCC therapists participated in phase 1 and phase 3 focus groups to obtain design specifications for which clinically meaningful outcomes AICF should capture and provide real-time analytics for, as well as the pros and cons after experiencing AICF clinically. All therapists who responded to our request to participate were involved in the study and are experienced in their field. In addition to the individual emotion tracking feature, the therapists expressed interest in tracking group processes with a particular emphasis on group cohesion. Therapists described group cohesion as a high frequency of posting by members with a sense of interconnectedness through replying to others. A successful group session results in members feeling supported and acknowledged by other group members. The results herein this manuscript excluded the results of these focus groups as they were published elsewhere.

#### Scoring Guide Development

A literature-based guide was developed to ensure that group cohesion statements were consistently identified and annotated by the human team.

Group cohesion is the sense of warmth, acceptance, support, and belongingness to the members [5], a sense of closeness, and participation [30]. It is measured by statements that reflect a sense of belonging and support in the group.

This belonging and support could be expressed with the statement themes below [31]. The following examples were from the CCC chat training data.

1. Reassurance or encouragement between peers
2. Expressing support or feeling supported
3. Deepening emotional disclosure and trust
4. A sense of belonging
5. Gratitude for the group
6. Finding shared experiences and commonalities
7. Looking forward to future sessions or connecting outside of the group
8. Reflecting on the positive aspects of the group

#### Creating Training Data

To train AICF to identify group cohesion, 1000 examples of cohesive statements from 10 OSG sessions were annotated by 2 human group therapists (EW and JH). These annotated examples were used for training the algorithm.

### Algorithm Development

#### Feature Selection of Group Cohesion Expressions

First, a corpus of CCC chat sessions (~80,000 messages) was used to train a word embedding model using Word2Vec using the Gensim library in Python (gensim.models.Word2Vec [documents, size=100, window=10, min_count=2, workers=10]). This enabled the creation of a vector representation for each word in the corpus. This positioned semantically similar expressions in closer proximity to generate contexts of cancer OSG discussion. Second, to expand the group cohesion mentions, the annotated samples were fed into the trained Word2Vec model as inputs to query for neighboring words. This resulted in a set of semantically similar, contextually relevant group cohesion expressions. This enabled AICF to identify statements representing group cohesion, including keywords such as “us,” “we,” and “our group,” as well as themes such as expressing gratitude, eagerness to attend upcoming group sessions, chatting outside of group time, mutual support, and a sense of belonging.

#### Training the Classifiers of Group Cohesion

To produce the probability of each post containing group cohesion, 3 models, multinomial naive Bayes, logistic regression, and multilayer perceptron (MLP) classifier with the group cohesion features selected were trained using the training data set.

Before training the classifier, a series of feature engineering steps were followed. Feature engineering is the process of creating features by extracting information from the data. For this purpose, the term frequency–inverse document frequency (TF-IDF) approach was used. TF-IDF is a statistical measure that evaluates how relevant a word is to a document in a collection of documents. It is performed by multiplying the term frequency and inverse document frequency of the word across a set of documents. In this classification task, the TF-IDF vectorizer was used with a limit of 5000 words capturing both unigrams (single words) and bi-grams (2 words that occur together). Next, the vectorizer was applied to the preprocessed training data set.

Once the data set was transformed, it was used to train multiple classifiers; naïve Bayes, random forest, support vector machine (SVM), multilayer perceptron (MLP), and logistic regression models. The objective of training multiple classifiers was to increase the performance of the final classification by incorporating multiple high-performing classifiers. This technique is called “soft voting,” which is an ensemble machine learning technique that combines predictions from multiple models. Table 1 shows the *F*_1_-scores of the trained classifiers.

**Table 1:** The F1-scores of trained classifiers.

Classifier	*F*_1_-score
Support vector machine	0.63
Naïve Bayes	0.79
Multilayer perceptron	0.77
Random forest	0.72
Logistic regression	0.82

#### Group Cohesion Score Calculation

Based on this, the top 3 classifiers were selected: naïve Bayes, MLP, and logistic regression. The outcomes of all 3 classifiers were used to make the final prediction. If a post is classified into the same label by 2 of the 3 classifiers, then the output label is used as the final outcome. A confidence value was generated for each classification based on the weighted *F*_1_-scores of each classifier. The average *F*_1_-score using 3 classifiers was 0.8.

**Figure 1:**
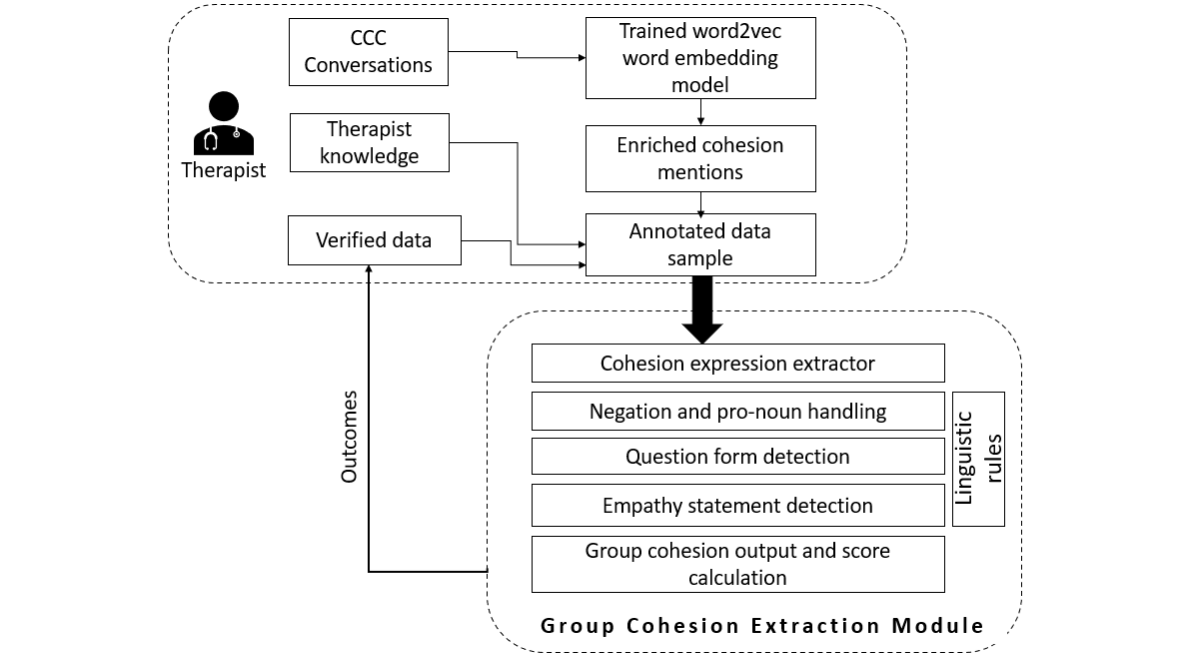
Process of group cohesion extraction. CCC: Cancer Chat Canada.

In order to improve the performance of the model, an active learning approach [32] was used where human input is used as feedback to fine-tune the models. Therapists examined 20% (6797/34048) of the outputs using a confusion matrix (see Active Learning via Human Scoring section). The scoring results were then used as a feedback loop to improve the list of keywords of queried expressions. Lastly, to fine-tune group cohesion extraction, linguistic rules were hand-coded to handle exceptions such as past tense and empathetic questions by participants (Figure 1).

#### Linguistic Rules for Group Cohesion Score Adjustment

The following rules were added after the first round of scoring based on therapists’ feedback:

1. Intensifiers: We have used the intensifiers from a pretrained library, Valence Aware Dictionary and Sentiment Reasoner (VADER; [33], which considers intensity boosters such as “very” and “so much” to enhance the valence.
2. Past tense (in the part-of-speech tagging via the NLTK Python library): The score would be multiplied by 0.5 if past tense was present, as the event had happened in the past, we assume that the effect of the event on the person would subside.
3. Negation: The calculated cohesion score would be set to zero in case of a negation expression.
4. First-person tagging: This was set to be “False” if second or third person pronouns were found.
5. Empathy: If an empathy statement were found, then the calculated group cohesion score would be doubled to denote the intensity.

Finally, an aggregated score of group cohesion (*β*) was calculated for specific time intervals using the following formula:



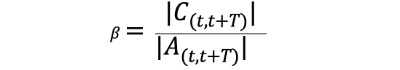



where *β* is the group cohesion score; *T* is the specified time interval (30 minutes); *A*(*t*,*t*+*T*) is the set of all posts the occurred during the time *t* to *t*+*T*; and *C*(*t*,*t*+*T*) is the set of cohesion mentioned posts that occurred during the time *t* to *t*+*T*.

A group cohesion score was displayed and updated at 30-minute intervals on the 90-minute timeline in a real-time dashboard for therapists.

#### Active Learning via Human Scoring

Outputs were scored by undergraduate students (responsible for basic emotions), graduate students, and clinical experts (responsible for clinical and process outcomes). The team scored 20% of the output to inform AICF development, which was improved in light of the human scoring results. The updated AICF was run on the data of a new OSG (test data). Each AICF version was saved before training with new data. The team scored the output using definitions or examples from well-established psychometric measures such as the Group Cohesiveness Scale and Group Openness and Cohesion Questionnaire. A confusion matrix was used to score AICF outputs. The scoring process was based on recall, precision, and *F*_1_ measures. Scorers’ feedback using their domain expertise was used to improve AICF’s performance until it achieved an *F*_1_-score of 80% before deploying in real-time OSG for beta-testing [34].

#### LIWC Evaluation

The Linguistic Inquiry Word Count (LIWC) software [35], considered the gold standard of psychology-based NLP, was used as a validation tool. LIWC reads a given text and calculates the percentage of total words in the text that match each of the LIWC dictionary categories. We tried to capture the concept of group cohesion using multiple LIWC dictionary categories: “we,” “positive emotion,” “family,” “friend,” and “affiliation” as the measurement criteria. We classified the text as an instance of group cohesion when at least 3 out of 5 criteria were met.

### Ethical Considerations

The study protocol including the human participant recruitment method was approved by the University Health Network Research Ethics Board (confirmation number: UHN REB#18-5354). All identifiable information was removed from the quotes in this report. Participants were compensated with a CAD (73.34 USD) gift card upon the completion of the focus group.

## Results

The results herein only focus on the human evaluation of the AICF system and its ability to detect group cohesion. We compared AICF to LIWC using human judgment using the confusion matrix and *F*_1_-score to measure accuracy and precision. AICF was run on 34,048 messages of CCC chat history to generate outputs for human scoring. Every fifth message was scored, totaling 6797 messages (20%). The precision, recall, and *F*_1_-scores are reported in Table 1 and show that logistic regression, followed by naive Bayes and MLP classifiers performed the best.

In this first round, AICF missed a high number of group cohesive statements (Table 2).

All scored statements were incorporated into AICF for improvement. In the second round, the team checked another 296 of 1208 messages (20%) from a separate set of CCC group conversations. AICF was able to improve the false-negative rate (recall) from 0.52 to 0.70.

We also ran LIWC on new OSG data (12,034 messages) from the CCC platform. Precision, recall, and *F*_1_-scores are listed in Table 3.

Within the “true positive” instances identified by AICF in agreement with the human scorers, several thematic categories and keywords emerged. They closely align with established measures of group cohesion [31], including expressions of support or a sense of belonging (Table 2). Moreover, some keywords consistently emerged within the true-positive statement classifications (eg, “we,” “us,” “our,” “group,” “support”). Among the false-positive identifications, it was typically due to a missed subtle negation within the sentence or when a participant wrote about a supportive person or activity from outside the group (Table 2).

Where AICF missed a classification of group cohesion (ie, a false negative), it was typically also due to nuanced conversational features on which it had not yet been trained, such as local expressions or idioms, supportive responses to others or statements missing identified group cohesion keywords (such as “we,” “us,” and “our”; Table 2). These correct and incorrect classifications were used to refine AICF detection of group cohesion as the algorithm progresses in development.

**Table 2:** Themes, keywords, and examples of AICF^a^ outputs.

Themes		Examples
**True-positive themes**		
	Reassurance among peers	“Good for you, that must have been really difficult to do”
	Expressing support/feeling supported	“sending hugs”; “thanks everyone for your support <3”
	Deeping emotional disclosure and trust	“At my last treatment, I felt really scared, I didn’t want to tell my family this. But I can tell this group”
	Sense of belonging	“I am glad to be part of this group”
	Gratitude for the group and peers	“Thank you everyone, this is such a great group.”
	Shared experiences	“It is amazing how much we all have in common”
	Reflecting on the positive aspects of the group	“This group has been a great resource venue for meeting and I will continue down the road with fond memories of the time spent here”
	Anticipating future groups/chats	“I can’t wait to chat with you all next week”
	Keywords: we, us, our, you all/all of you, thanks / thank you, time, chat, group, support, miss	“we are friends”; “thinking of you all!”; “time went by fast!”;“A very good chat session”; “I’m so glad I found this group”; “thank you for your support and input”; “I am going to miss this group”
**False-positive themes**		
	False detection of “we” or “us” when it is not indicative of group cohesion	“When I mentioned that last week, when we all said what our situations were, no one even acknowledged it. I was very hurt by that”
	Talking about support from nongroup members	“When I put my things in order I involved my children and as challenging and emotional as it was it made it easier for me and for them - we laughed and cried but it really made me feel supported”
**False-negative themes**		
	Idioms/expressions	“been there, am there... got the t-shirt”; “I hope you don't think you've been placed in a hot seat. It's just that I missed you and worried about you.”; “we are joining our circle“
	Missed detection of providing empathy, encouragement, appreciation, and support to other members	“I am with you, be strong please”; “Are you going to be alright? I want you to know how much I appreciate your presence.”
	Missed detection of agreeing with or relating to others’ stories	“I can relate”; “I feel the same way”;
	Missed statements because of lack of “we” language	“I am sure there will be a sense of connection, so much sharing already”; “I am sure I will think of you often”

^a^AICF: Artificial Intelligence–based Co-Facilitator.

**Table 3:** Artificial Intelligence–based Co-Facilitator performance evaluation for identification of group cohesion.

Scoring round/method	Precision	Recall	*F*_1_-score
First	0.99	0.52	0.68
Second	0.98	0.70	0.82
Linguistic Inquiry and Word Count	0.36	0.23	0.28

## Discussion

### Principal Results

AICF, an ensemble of NLP and machine learning algorithms combined with annotation and human scoring, offers a novel way of measuring the group cohesion changes for each group member and alerting the therapist of these changes in real time. This affords therapists the opportunity to allocate their attention and resources for effective facilitation. The objective was to determine whether AICF can detect group cohesion beyond the first-person plural pronunciation use. The findings indicate that it is feasible to measure group cohesion in text-based complex human interactions using AICF. The level of congruency with human scoring suggests that it can be a helpful tool to therapists in improving the group cohesion outcome.

This study has opened an avenue to person-centered and process-outcome research using AI combined with human inputs to improve the quality of care, which otherwise is a labor-intensive research process. Initially, after being trained with 1000 annotated group cohesion statements processed by word embeddings and the domain expertise from therapists, AICF was able to achieve reasonable *F*_1_, precision, and recall scores. Furthermore, training the algorithm using only word embeddings allows AICF to identify the various cohesion themes that emerged, which are consistent with previous research 34]. These themes include expressing support, reassurance, a sense of belonging, trust, deepening emotional disclosure, gratitude, remarking on shared experiences, reflecting on positive aspects of the group, and anticipating future chats. The findings suggest that training AICF to monitor therapeutic responses in web-based care is promising.

When combined with the human scoring examples in the algorithm, as little as 20% of the outputs, AICF obtained a high *F*_1_-score. The human rater detected both false-positive examples (eg, “Just have to find what works for you, I listen to a lot of audible books while I do chores, it's a mental distraction and really helps me”) and false-negative examples (eg, “Thanks so much to all of you, for being in this moment. You've helped me get ready for yet another week.”). These examples contributed to the rule-based algorithms as a second layer of analysis. While precision values remained relatively low in both rounds (0.99 vs 0.98), the recall value improved from 0.52 to 0.70 due to a reduction in false-negative classifications. These increases strongly suggest that a continuous effort to train AICF using human input can lead to a higher level of accuracy in detecting group cohesion.

After running LIWC on a test data set, its performance was evaluated by a human scorer. The precision, recall, and *F*_1_-scores were lower compared to the performance of AICF. Unlike AICF, which is capable of identifying group cohesion expressions and idioms, LIWC is programmed to identify certain keywords. For a false-negative example, LIWC was unable to detect the following quote as an instance of group cohesion due to the lack of the keyword “we”: “I feel like I’ve suddenly inherited a whole group of sisters.” Another instance of an LIWC false positive was that LIWC dictionary categories “family,” “positive emotion,” and “affiliation” falsely detected group cohesion from this quote: “My husband has helped me see that it isn't something I did, or who I am.”

### Comparison With Prior Work

This study successfully trained a machine learning system to detect cohesive statements in contrast to qualitative content analysis, which tends to be onerous and prone to human errors when dealing with large amounts of data [11]. Emerging computer programs such as Discourse Attributes Analysis Program (DAAP) [36] and LIWC [35,37] offer an iterative psycholinguistic approach to coding transcripts of psychotherapy for therapeutic moments [18,21]. For example, DAAP is based on a weighted dictionary that assigns weights to different words instead of solely detecting them as belonging to various categories where all matching words contribute equally to the generated scores. This method allows for greater accuracy in measuring different concepts compared to human coding while processing large amounts of data. However, these weighted dictionary approaches can be limited by a fixed number of instances that can be detected, and only one keyword can be considered in each matching rather than taking contexts into account. Additionally, they do not consider emerging words, phrases, idioms of expressions, word order, negation, and context-dependent factors, as well as their post hoc nature [19]. In this study, the word embedding approach was used to create contextual variables from the keywords to successfully detect a reasonably broad definition of cohesiveness. Thus, work will continue toward improving the accuracy of AICF in upcoming OSG sessions.

### Limitations

AICF is based on a previously trained ensemble called Patient-Reported Information Multidimensional Exploration (PRIME) that was primarily trained on Australian web-based forum data [29]. Thus, Canadians may have used expressions or idioms that were unfamiliar to the original PRIME system and, therefore, not detected (eg, “My head is swirling” to describe feeling overwhelmed or “the clock is ticking” to describe an impending end of life). The local idioms and expressions were handled by the rule-based approach; ideally, AICF would be (re)trained with a large amount of local data in order to capture such idioms and expressions.

Currently, the interactional nature of the statement is not incorporated into AICF, including responses to other members’ or therapists’ statements. Furthermore, AICF cannot consistently distinguish whether participants are speaking about the group or about people outside of the group. When data accumulate, this distinction will become more obvious and refine AICF’s detection ability within the context of an OSG.

The performance of AICF’s group cohesion classification was evaluated in comparison to scores by 2 human experts, whose scoring was guided by the same criteria. However, given the nuanced nature of a group process like cohesion, there was still an element of personal judgment and openness to interpretation in the statements. Finally, emojis were not considered in the algorithm; future studies need to incorporate them as expressions of group cohesion.

### Future Directions

ICF has been running in the background on 3 CCC groups and will soon be deployed for beta-testing on 10-12 groups. Participants will be filling out a survey package that includes the psychometrically validated questionnaire that tabs group cohesion for further validation. For algorithm development, sequencing the emotions expressed by each participant will be explored to capture more accurate emotional profiles.

The use of large language models (LLMs), such as ChatGPT, has revolutionized natural language understanding in the field of affective computing. Research suggests that an LLM called ROBERTa [38] has been equipped with emotion knowledge that contains 14 human conceptual attributes of emotions, including 2 affective, 6 appraisals, and 6 basic emotions. Future work will incorporate LLMs into our system to enhance AICF’s ability to detect group cohesion and other significant clinical outcomes. For example, the LLM has already understood the syntactic difference between first-person and third-person pronunciation uses and their contexts. Combining both of these emotional attributes and syntax, we are able to better formulate an equation to calculate the tendency of a writer to be self-focused or other-focused. This will truly improve the accuracy and precision of group cohesion detection.

Lastly, In this study, 5 LIWC dictionary categories were used to capture the concept of group cohesion. Future studies may test whether there is a way that will improve the performance of group cohesion prediction using LIWC by (1) adding more categories, (2) reducing some categories, and (3) adding weighting to each criterion.

AICF will explore ways to measure multiple processes comprising group climate, including the level of participation, expression of emotion, signs of cohesion, avoidance, and therapeutic factors such as conflict, altruism, universality, interpersonal learning input and output, catharsis, identification, self-understanding, and instillation of hope [5,9,31]. If successful, AICF will be applied alongside the mobile health chatbot technology to provide a scalable, automated monitoring and referral system that screens users for specific symptoms, recommends individualized web-based and community resources, tracks each user’s psychological outcomes through, and refers them to local therapists when necessary.

### Conclusions

Optimal OSG delivery requires rapid alerts for therapists to effectively monitor markers of positive and negative responses within the group. This study has demonstrated that advanced machine learning algorithms combined with human inputs can reasonably detect the clinically meaningful indicator of group cohesion in OSGs. Future research in utilizing LLMs in AICF could enhance the capabilities in understanding the context, given the capability of creating a highly customized model in a short time. Therefore, AICF has the potential to assist therapists by highlighting issues that are amenable to intervention in real time, which allows therapists to provide greater levels of individualized support.

## Abbreviations

AICF: Artificial Intelligence–based Co-Facilitator
CCC: Cancer Chat Canada
DAAP: Discourse Attributes Analysis Program
LIWC: Linguistic Inquiry and Word Count
NLP: natural language processing
OSG: online support group
PRIME: Patient-Reported Information Multidimensional Exploration
TF-IDF: term frequency–inverse document frequency
VADER: Valence Aware Dictionary and Sentiment Reasoner

## Data Availability

The data sets generated during or analyzed during this study are not publicly available due to the presence of private health information but are available from the corresponding author on reasonable request.

## References

[1] Gratzer D, Goldbloom D (2016). Making evidence-based psychotherapy more accessible in Canada. Can J Psychiatry.

[2] Male DA, Fergus KD, Stephen JE (2017). Professional online support group facilitators: guarantors of maximal group utility. Int J Group Psychother.

[3] McCaughan E, Parahoo K, Hueter I, Northouse L, Bradbury I (2017). Online support groups for women with breast cancer. Cochrane Database Syst Rev.

[4] van Uden-Kraan CF, Drossaert CHC, Taal E, Shaw BR, Seydel ER, van de Laar MAFJ (2008). Empowering processes and outcomes of participation in online support groups for patients with breast cancer, arthritis, or fibromyalgia. Qual Health Res.

[5] Yalom ID (2005). The Theory and practice of group psychotherapy, 5th ed. The Theory and Practice of Group Psychotherapy.

[6] Anderson WG, Alexander SC, Rodriguez KL, Jeffreys AS, Olsen MK, Pollak KI, Tulsky JA, Arnold RM (2008). "What concerns me is..." Expression of emotion by advanced cancer patients during outpatient visits. Support Care Cancer.

[7] Roos J, Werbart A (2013). Therapist and relationship factors influencing dropout from individual psychotherapy: a literature review. Psychother Res.

[8] Clarke G, Eubanks D, Reid E, Kelleher C, O'Connor E, DeBar LL, Lynch F, Nunley S, Gullion C (2005). Overcoming Depression on the Internet (ODIN) (2): a randomized trial of a self-help depression skills program with reminders. J Med Internet Res.

[9] Johnson JE, Burlingame GM, Olsen JA, Davies DR, Gleave RL (2005). Group climate, cohesion, alliance, and empathy in group psychotherapy: multilevel structural equation models. J. Couns. Psychol.

[10] Treadwell T, Lavertue N, Kumar VK, Veeraraghavan V (2001). The group cohesion scale-revised: reliability and validity. Int. J. Action Methods Psychodrama Ski. Train. Role Play.

[11] Sierra Hernandez CAS, Piper WE, Ogrodniczuk JS, Joyce AS, Weideman R (2016). Use of referential language in short-term group psychotherapy for complicated grief. Group Dyn. Theory Res. Pract. Wash.

[12] Fineberg SK, Leavitt J, Deutsch-Link S, Dealy S, Landry CD, Pirruccio K, Shea S, Trent S, Cecchi G, Corlett PR (2016). Self-reference in psychosis and depression: a language marker of illness. Psychol Med.

[13] Pennebaker JW, King LA (1999). Linguistic styles: language use as an individual difference. J Pers Soc Psychol.

[14] Tackman AM, Sbarra DA, Carey AL, Donnellan MB, Horn AB, Holtzman NS, Edwards TS, Pennebaker JW, Mehl MR (2019). Depression, negative emotionality, and self-referential language: A multi-lab, multi-measure, and multi-language-task research synthesis. J Pers Soc Psychol.

[15] Swol LMV, Kane AA Language and group processes: an integrative, interdisciplinary review. Small Group Res.

[16] Piper WE, McCallum WE (2000). The psychodynamic work and object rating system. APA.

[17] (1991). dtSearch.

[18] Brockmeyer T, Zimmermann J, Kulessa D, Hautzinger M, Bents H, Friederich H, Herzog W, Backenstrass M (2015). Me, myself, and I: self-referent word use as an indicator of self-focused attention in relation to depression and anxiety. Front Psychol.

[19] Alpers GW, Winzelberg AJ, Classen C, Roberts H, Dev P, Koopman C, Barr Taylor C (2005). Evaluation of computerized text analysis in an Internet breast cancer support group. Comput. Hum. Behav.

[20] Lieberman MA, Wizlenberg A, Golant M, Di Minno M (2005). The impact of group composition on Internet support groups: Homogeneous versus heterogeneous Parkinson's groups. Group Dyn. Theory Res. Pract.

[21] Mariani R, Maskit B, Bucci W, De Coro A (2013). Linguistic measures of the referential process in psychodynamic treatment: the English and Italian versions. Psychother Res.

[22] Yvonne Leung, Elise Wouterloot, Achini Adikari, Graeme Hirst, Daswin De Silva, Jiahui Wong, Jacqueline Bender, Mathew Gancarz, David Gratzer, Damminda Alahakoon, Mary Jane Esplen (2021). Natural language processing–based virtual cofacilitator for online cancer support groups: protocol for an algorithm development and validation study. JMIR research protocols.

[23] Robert Plutchik (1980). Chapter 1 - A general psychoevolutionary theory of emotion. Theories of Emotion.

[24] Leung YW, Wouterloot E, Adikari A, Hirst G, de Silva D, Wong J, Bender JL, Gancarz M, Gratzer D, Alahakoon D, Esplen MJ (2021). Natural language processing-based virtual cofacilitator for online cancer support groups: protocol for an algorithm development and validation study. JMIR Res Protoc.

[25] Leung YW, Park B, Heo R, Adikari A, Chackochan S, Wong J, Alie E, Gancarz M, Kacala M, Hirst G, de Silva D, French L, Bender J, Mishna F, Gratzer D, Alahakoon D, Esplen M (2022). Providing care beyond therapy sessions with a natural language processing-based recommender system that identifies cancer patients who experience psychosocial challenges and provides self-care support: pilot study. JMIR Cancer.

[26] Leung YW, Park B, Heo R, Adikari A, Chackochan S, Wong J, Alie E, Gancarz M, Kacala M, Hirst G, de Silva D, French L, Bender J, Mishna F, Gratzer D, Alahakoon D, Esplen MJ (2022). The Patient-Reported Information Multidimensional Exploration (PRIME) framework for investigating emotions and other factors of prostate cancer patients with low intermediate risk based on online cancer support group discussions. JMIR Cancer.

[27] Hu J, Jin F, Zhang G, Wang J, Yang Y (2017). A user profile modeling method based on Word2Vec.

[28] De Silva D, Ranasinghe W, Bandaragoda T, Adikari A, Mills N, Iddamalgoda L, Alahakoon D, Lawrentschuk N, Persad R, Osipov E, Gray R, Bolton D (2018). Machine learning to support social media empowered patients in cancer care and cancer treatment decisions. PLoS One.

[29] Ranasinghe W, Bandaragoda T, De Silva D, Alahakoon D (2017). A novel framework for automated, intelligent extraction and analysis of online support group discussions for cancer related outcomes. BJU Int.

[30] Dies RR, MacKenzie KR (1983). Advances in group psychotherapy: integrating research and practice. AGPA.

[31] Borek AJ (2019). Developing and applying a framework to understand mechanisms of action in group-based, behaviour change interventions: the MAGI mixed-methods study. Effic. Mech. Eval.

[32] Zeng J, Liang J, Wang X, Li L, Zeng D, Luo B, Cheng L, Wu ZG, Li H, Li C (2024). A two-stage active learning algorithm for NLP based on feature mixing. Neural Information Processing. ICONIP 2023 Communications in Computer and Information Science.

[33] Hutto C, Gilbert E (2014). VADER: A parsimonious rule-based model for sentiment analysis of social media text. ICWSM.

[34] Cota AA, Dion KL, Evans CR (2016). A Reexamination of the Structure of the Gross Cohesiveness Scale. Educ. Psychol. Meas.

[35] Tausczik YR, Pennebaker JW (2009). The psychological meaning of words: LIWC and computerized text analysis methods. J. Lang. Soc. Psychol.

[36] Maskit Bernard (2021). Overview of Computer Measures of the Referential Process. J Psycholinguist Res.

[37] Pennebaker JW, Mayne TJ, Francis ME (1997). Linguistic predictors of adaptive bereavement. J Pers Soc Psychol.

[38] Li M (2023). Human emotion knowledge representation emerges in large language model and supports discrete emotion inference.

